# Reflux Recurrence After Laparoscopic Fundoplication for Nonerosive Gastroesophageal Reflux Disease

**DOI:** 10.1001/jamanetworkopen.2025.17754

**Published:** 2025-06-30

**Authors:** Dag Holmberg, Julia Bielik, Giola Santoni, Eivind Ness-Jensen, My von Euler-Chelpin, Joonas H. Kauppila, Jesper Lagergren

**Affiliations:** 1 Department of Molecular Medicine and Surgery, Karolinska Institutet and Karolinska University Hospital, Stockholm, Sweden; 2 Department of Public Health and Nursing, Norwegian University of Science and Technology, Trondheim, Norway; 3 Medical Department, Levanger Hospital, Nord-Trøndelag Hospital Trust, Levanger, Norway; 4 Department of Public Health, University of Copenhagen, Copenhagen, Denmark; 5 Department of Surgery, Oulu University Hospital and University of Oulu, Oulu, Finland; 6 School of Cancer and Pharmaceutical Sciences, King’s College London, London, United Kingdom

## Abstract

**Importance:**

Gastroesophageal reflux disease (GERD) is very common in high-income countries. Nonerosive GERD is increasingly being considered a different entity from erosive GERD, with a more benign disease course but less responsiveness to antireflux medication. Whether nonerosive GERD responds less well than erosive GERD to antireflux surgery is unclear.

**Objective:**

To assess whether patients with nonerosive GERD retain more reflux symptoms after antireflux surgery than patients with erosive GERD.

**Design, Setting, and Participants:**

This population-based cohort study included all patients in Finland and Sweden who underwent primary laparoscopic fundoplication for GERD between January 1, 1996, and December 31, 2019. Statistical analysis was conducted from March to April 2024.

**Exposure:**

Patients with nonerosive GERD (ie, no erosive esophagitis or Barrett esophagus detected during preoperative endoscopy) were compared with patients with erosive GERD (ie, erosive esophagitis detected during preoperative endoscopy).

**Main Outcome and Measures:**

The main outcome was reflux recurrence, defined as 6 months or more of postoperative antireflux medication or secondary antireflux surgery. Poisson regression provided hazard ratios (HRs) with 95% CIs, adjusted for sex, age, comorbidity, hospital volume of antireflux surgery, calendar year, and country.

**Results:**

Of 6194 patients (median age, 53 years [IQR, 42-62 years]; 3310 women [53.4%]) who underwent primary fundoplication, 2700 (43.6%) received a diagnosis of nonerosive GERD, and 3494 (56.4%) received a diagnosis of erosive GERD. During up to 23 years of follow-up (range, 0-23 years; median, 8.8 person-years [IQR, 4.3-13.5 person-years]), the frequency of reflux recurrence was similar among patients with nonerosive GERD (17.1% [461 of 2700]) and those with erosive GERD (17.1% [596 of 3494]). Patients with nonerosive GERD had a similar overall risk of reflux recurrence as patients with erosive GERD (adjusted HR, 0.98; 95% CI, 0.87-1.11) and a similar risk of reflux recurrence when studying recurrence by antireflux medication (HR, 1.04; 95% CI, 0.90-1.21) and secondary antireflux surgery (HR, 0.91; 95% CI, 0.75-1.10) separately. There were no differences in HRs between various follow-up categories after fundoplication or in analyses stratified by the 6 variables included in the multivariable model.

**Conclusions and Relevance:**

This cohort study of patients who underwent primary laparoscopic fundoplication suggests that the risk of reflux recurrence was similar among patients with nonerosive GERD and those with erosive GERD. This finding is in contrast with evidence showing that nonerosive GERD responds less well to antireflux medication. Thus, the absence of erosive GERD detected by an upper endoscopy may not be used as an argument for abstaining from antireflux surgery.

## Key Points

**Question:**

Is laparoscopic fundoplication associated with increased risk of reflux recurrence among patients with nonerosive gastroesophageal reflux disease (GERD) compared with erosive GERD?

**Findings:**

In a binational population-based cohort study including 6194 patients who received laparoscopic fundoplication and were followed up for up to 23 years without losses to follow-up, the cumulative incidence of reflux recurrence was 17% for patients with nonerosive GERD and those with erosive GERD.

**Meaning:**

This study suggests that the risk of reflux recurrence after laparoscopic fundoplication may be similar among patients with nonerosive and erosive GERD.

## Teaser

This cohort study assesses whether patients with nonerosive gastroesophageal reflux disease (GERD) retain more reflux symptoms after laparoscopic fundoplication than patients with erosive GERD.

## Introduction

Gastroesophageal reflux disease (GERD), typically presenting as repeated and troublesome symptoms of heartburn or regurgitation of gastric contents, occurs in 15% to 25% of adult individuals in high-income countries.^1,2,3^ Objective manifestations of GERD include mucosal injury visible by upper endoscopy (ie, esophagitis or Barrett esophagus), termed *erosive GERD*, or pathologic esophageal pH measurements.^4^ Typical GERD symptoms combined with a normal upper endoscopic finding and a pathologic pH measurement is termed *nonerosive GERD*.^4^ Patients with nonerosive GERD are more often women, younger, and rarely develop GERD-related complications, such as Barrett esophagus or esophageal adenocarcinoma, in contrast to patients with erosive GERD.^5,6^ The predominant treatment of GERD is medication with a proton pump inhibitor. Antireflux surgery, primarily laparoscopic fundoplication, is a less-common option that may help the 30% of patients who continue to experience reflux symptoms even after taking antireflux medication.^7,8^ Patients with nonerosive GERD often respond poorly to antireflux medication, in contrast to patients with erosive GERD, often leading to persistent or recurring reflux symptoms despite treatment.^9,10,11^ It is unclear how well laparoscopic fundoplication controls reflux symptoms among patients with nonerosive GERD compared with those with erosive GERD.

We hypothesized that the effectiveness of fundoplication would be lower among patients with nonerosive GERD, similar to the situation for antireflux medication. This hypothesis was tested in a large and unselected cohort of patients who had undergone primary laparoscopic fundoplication.

## Methods

### Design

This was a population-based cohort study conducted between January 1, 1996, and December 31, 2018, in Finland and between July 1, 2005, and December 31, 2019, in Sweden. Patients eligible for inclusion were adults (>18 years) with GERD (eTable 1 in Supplement 1) who had undergone primary laparoscopic fundoplication and were preoperatively examined with upper endoscopy in the year prior to surgery (eTable 2 in Supplement 1). Apart from endoscopy, standard workup for laparoscopic fundoplication in Finland and Sweden includes pH measurements demonstrating pathologic acid reflux and manometry excluding other diagnoses. Patients were identified from the nationwide patient registries in Finland and Sweden, which prospectively collect individual-level data on all diagnoses (including GERD), diagnostic examinations (including endoscopy), and surgical procedures (including fundoplication) from all in-hospital and hospital-based outpatient health care facilities.^12^ Data from the Finnish and Swedish patient registries have been extensively validated for their use in research, and the coverage and specificity are high.^13,14^ Patients having undergone any surgery of the esophagus or with a diagnosis of Barrett esophagus before the primary laparoscopic fundoplication were not eligible (eTable 3 in Supplement 1). The study was approved by the National Board of Health and Welfare in Sweden and the National Institute for Health and Welfare and Statistics Finland. Patient consent was waived by the review boards in each country due to the registry-based design of the study and because data were anonymized. This study followed the Strengthening the Reporting of Observational Studies in Epidemiology (STROBE) reporting guideline for cohort studies.

### Exposure

The exposed group of patients had nonerosive GERD (ie, a diagnosis of GERD without esophagitis recorded during upper endoscopy before the fundoplication). The comparison (nonexposed) group comprised patients with erosive GERD (ie, reflux esophagitis recorded during endoscopy conducted before the fundoplication). The exposure information came from the national patient registries.

### Outcomes

The primary outcome was the recurrence of reflux, defined as either the use of antireflux medication with a proton pump inhibitor (Anatomical Therapeutic Code [ATC] code A02BC) or a histamine-2 receptor antagonist (ATC code A02BA) for 6 months or more after the primary laparoscopic fundoplication or after undergoing secondary antireflux surgery (laparoscopic or open) (eTable 4 in Supplement 1). Reflux recurrence after primary laparoscopic fundoplication in Sweden and England has also been studied using these criteria.^15,16^ Secondary outcomes were analyzed separately for antireflux medication and secondary antireflux surgery. Antireflux medication data were retrieved from the national prescribed drug registries in Finland and Sweden, which prospectively collect data on all prescribed and dispensed medications in the 2 countries.^17^ Information on secondary antireflux surgery was retrieved from the patient registries in Finland and Sweden.

### Confounders

We examined 6 potential confounders: sex (male or female), age (continuous), comorbidities (Charlson Comorbidity Index score of 0, 1, or ≥2), annual hospital volume of laparoscopic fundoplication (4 equal-sized groups [ie, quartiles]), calendar year (continuous), and country (Finland or Sweden). The most validated and updated edition of the Charlson Comorbidity Index was used (eTable 5 in Supplement 1).^18^ Data for all confounders were retrieved from the patient registries and indexed at the date of the primary laparoscopic fundoplication.

### Statistical Analysis

Statistical analysis was conducted from March to April 2024. Follow-up started from the date of primary fundoplication and ended at the date of reflux recurrence, diagnosis of Barrett esophagus or esophageal cancer (eTable 6 in Supplement 1), death, or the end of the study period, whichever occurred first. Time of outcome event was defined as the date of dispensation of 180 or more defined daily doses of antireflux medication or the date of secondary antireflux surgery, whichever occurred first. Poisson regression, with time to event finely divided into small intervals, modeled with cubic splines of time with 4 knots, was used to calculate hazard ratios (HRs) with 95% CIs estimated using robust variance estimation methods. Apart from a crude model without any adjustments, a main model was adjusted for the 6 confounders with the aforementioned categories. Hazard ratios were analyzed for the entire follow-up (0-23 years) as well as for follow-up times categorized into 5 years or less, 6 to 10 years, and 10 years or more after fundoplication. Additional analyses were stratified by sex, age (above or below median), comorbidity (Charlson Comorbidity Index score of 0, 1 or ≥2), annual hospital volume (above or below median), calendar year (above or below median), and country (Finland or Sweden). The data management and statistical analysis followed a detailed and predefined study protocol and were conducted by a senior biostatistician (G.S.) using the statistical software Stata/MP, version 15.1 (StataCorp LLC). All *P* values were from 2-sided tests and results were deemed statistically significant at *P* < .05.

## Results

### Patients

The cohort included 6194 patients (median age, 53 years [IQR, 42-62 years]; 3310 women [53.4%] and 2884 men [46.6%]) who had undergone primary laparoscopic fundoplication for GERD (Table 1). The preoperative upper endoscopic findings characterized 2700 of these participants (43.6%) as having nonerosive GERD and 3494 (56.4%) as having erosive GERD. Patient characteristics were similarly distributed between the 2 groups, except for a higher frequency of women and a slightly higher median age in the nonerosive GERD group.

**Table 1.** Characteristics of Patients With Primary Laparoscopic Fundoplication for GERD in Finland and Sweden

Characteristic	Patients, No. (%)	
	Nonerosive (n = 2700)	Erosive (n = 3494)
Sex		
Female	1573 (58.3)	1737 (49.7)
Male	1127 (41.7)	1757 (50.3)
Age, median (IQR), y	54 (43-64)	51 (41-61)
Charlson Comorbidity Index score		
0	1668 (61.8)	2230 (63.8)
1	701 (26.0)	914 (26.2)
≥2	331 (12.3)	350 (10.0)
Annual hospital volume of laparoscopic fundoplication		
≤13	760 (28.2)	888 (25.4)
14-28	652 (24.2)	833 (23.9)
29-50	682 (25.3)	849 (24.3)
>50	606 (22.4)	924 (26.4)
Calendar year, median (IQR)	2010 (2005-2015)	2008 (2004-2013)
Country		
Finland	1831 (67.8)	2598 (74.4)
Sweden	869 (32.2)	896 (25.6)
Follow-up time, median (IQR), y	7.0 (2.9-12.3)	8.9 (4.0-13.3)
Recurrence of reflux	461 (17.1)	596 (17.1)
≥6 mo of Antireflux medication	320 (11.9)	410 (11.7)
Secondary antireflux surgery	190 (7.0)	230 (6.6)
Barrett esophagus	34 (1.3)	85 (2.4)
Esophageal or gastroesophageal junction cancer	3 (0.1)	8 (0.2)

Abbreviation: GERD, gastroesophageal reflux disease.

### Recurrence of Reflux

During follow-up (range, 0-23 years; median, 8.8 person-years [IQR, 4.3-13.5 person-years]), a total of 1057 patients (17.1%) had reflux recurrence (461 of 2700 [17.1%] with nonerosive GERD and 596 of 3494 [17.1%] with erosive GERD), of whom 730 (11.8%) used antireflux medication and 420 (6.8%) had secondary antireflux surgery (Table 1). Patients with nonerosive GERD had a similar overall risk of reflux recurrence as patients with erosive GERD (adjusted HR, 0.98; 95% CI, 0.87-1.11), and this lack of association was consistent across the 3 follow-up time periods (Table 2; Figure). There were no differences in the risk of reflux recurrence between nonerosive and erosive GERD in the analyses stratified by sex, age groups, comorbidity, calendar periods, or country (Table 2). Reflux recurrence was also similarly common among patients with nonerosive compared with patients with erosive GERD in higher-volume hospitals (adjusted HR, 0.92; 95% CI, 0.77-1.10) and in low-volume hospitals (adjusted HR, 1.04; 95% CI, 0.88-1.23) (Table 2). There were no differences in the risk of reflux recurrence among patients with nonerosive compared with patients with erosive GERD in separate analyses of antireflux medication as the single outcome (adjusted HR, 1.04; 95% CI, 0.90-1.21) or secondary antireflux surgery as the single outcome (adjusted HR, 0.91; 95% CI, 0.75-1.10).

**Table 2.** Risk of Reflux Recurrence After Primary Laparoscopic Fundoplication Among Patients With Nonerosive vs Erosive GERD in Finland and Sweden

Characteristic	Nonerosive		Erosive		HR (95%CI)		
					Erosive	Nonerosive	
	No. of person-years	No. of cases	No. of person-years	No. of cases		Crude	Adjusted^a^
Overall	21 705	461	31 645	596	1.00 [Reference]	1.09 (0.97-1.23)	0.98 (0.87-1.11)
Follow-up period, y							
≤5	10 530	342	14 584	401	1.00 [Reference]	1.18 (1.02-1.36)	1.00 (0.87-1.16)
6-10	6383	76	9980	121	1.00 [Reference]	0.98 (0.74-1.31)	0.95 (0.71-1.27)
>10	4792	43	7081	74	1.00 [Reference]	0.86 (0.59-1.25)	0.90 (0.62-1.31)
Sex							
Female	11 735	303	14 881	343	1.00 [Reference]	1.08 (0.92-1.26)	0.98 (0.84-1.14)
Male	9970	158	16 765	253	1.00 [Reference]	1.03 (0.85-1.26)	0.99 (0.81-1.20)
Age, y							
≤53	11 952	199	19 104	307	1.00 [Reference]	1.03 (0.86-1.23)	0.99 (0.83-1.18)
>53	9753	262	12 542	289	1.00 [Reference]	1.11 (0.94-1.31)	0.98 (0.83-1.16)
Charlson Comorbidity Index score							
0	15 265	249	21 926	317	1.00 [Reference]	1.12 (0.95-1.32)	1.05 (0.88-1.24)
1	4832	131	7546	198	1.00 [Reference]	0.97 (0.78-1.21)	0.84 (0.67-1.05)
≥2	1608	81	2174	81	1.00 [Reference]	1.23 (0.90-1.67)	1.06 (0.78-1.45)
Annual hospital volume of laparoscopic fundoplication							
≤28	8532	254	13 360	285	1.00 [Reference]	1.28 (1.08-1.52)	1.04 (0.88-1.23)
>28	13 172	207	18 286	311	1.00 [Reference]	0.93 (0.78-1.11)	0.92 (0.77-1.10)
Calendar year							
≤2009	15 550	212	24 887	337	1.00 [Reference]	1.02 (0.86-1.21)	1.03 (0.87-1.22)
>2009	6155	249	6759	259	1.00 [Reference]	1.02 (0.86-1.22)	0.92 (0.77-1.09)
Country							
Finland	16 948	245	25 419	389	1.00 [Reference]	0.94 (0.80-1.10)	0.91 (0.78-1.07)
Sweden	4757	216	6226	207	1.00 [Reference]	1.25 (1.03-1.51)	1.09 (0.90-1.33)

Abbreviations: GERD, gastroesophageal reflux disease; HR, hazard ratio.

^a^ Adjusted for sex, age, comorbidity, annual hospital volume of laparoscopic fundoplication, calendar year, and country.

**Figure.**
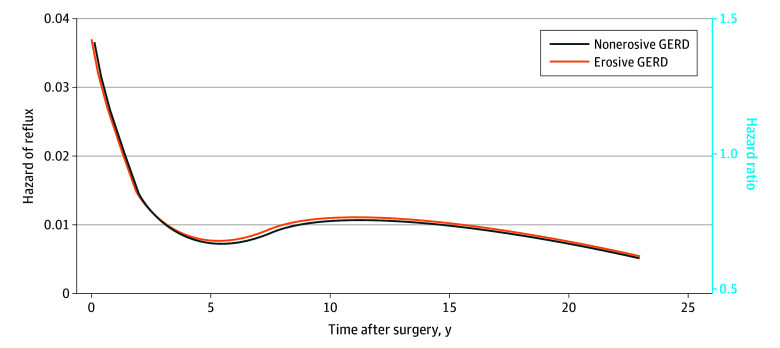
Recurrence Over Time After Primary Laparoscopic Fundoplication Hazard function of recurrence is shown over time after primary laparoscopic fundoplication among patients with nonerosive and erosive gastroesophageal reflux disease (GERD) as well as hazard ratio comparing nonerosive vs erosive GERD (solid dark blue line), including an adjusted model with no interaction between time and exposure.

## Discussion

This study found that recurrence of reflux after primary laparoscopic fundoplication was equally common among patients with nonerosive GERD as among patients with erosive GERD. The null association was consistent across follow-up periods and subgroups of sex, age, comorbidity, hospital volume, calendar year, and country, as well as in separate analyses of medication and secondary fundoplication as the outcome measure of reflux recurrence. To our knowledge, this is the largest study to date examining the risk of reflux recurrence after fundoplication among patients with nonerosive GERD.

Previous studies have provided contradictory results regarding the recurrence rate of reflux after fundoplication among patients with nonerosive vs erosive GERD. A Dutch cohort study compared 96 patients with nonerosive GERD with 117 patients with erosive GERD and found seemingly similar rates of relief of reflux symptoms (89% vs 96%), postoperative use of antireflux medication (21% vs 15%), and reintervention rates (15% vs 13%) within 5 years of surgery.^19^ However, the estimates had low statistical power. Other studies have reported on subjective outcomes of symptom control, with varying results, showing either poorer^20^ or equal symptom control^21,22,23,24,25^ after fundoplication among patients with nonerosive GERD compared with patients with erosive GERD. In contrast to the present study, previous studies had small sample sizes, short and incomplete follow-up, and were conducted in single centers. Due to selection bias and follow-up loss, these issues led to poor generalizability, low statistical power to detect differences between the comparison groups, and doubtful validity.

Nonerosive GERD is known to be difficult to treat medically. Patients often respond only partly or poorly to medications, leading to long-term on-demand use with unsatisfactory symptom relief. This study adds clinical data from 2 countries indicating that laparoscopic fundoplication effectively reduces GERD symptoms among patients with nonerosive GERD, who may respond less well to antireflux medication. The study also demonstrates the robustness of outcomes of laparoscopic fundoplication for nonerosive GERD among an unselected patient population, including surgery performed in low-volume hospitals. The results of this study should not be inferred to the entire population of patients with nonerosive GERD because patients selected for fundoplication represent a distinct and more well-defined subpopulation. They typically have more severe symptoms, often with incomplete response to antireflux medication, and undergo objective diagnostic workup demonstrating pathologic pH measurements that correlate well with the reflux symptoms.

### Strengths and Limitations

This study has some strengths, including the population-based design, which provided an unselected cohort of patients. The categorizations of nonerosive GERD and erosive GERD were based on objective endoscopic findings for all patients. The definition of GERD recurrence by resumption of regular antireflux medication or secondary antireflux surgery is a measurable and robust outcome.^15^ The use of merged data from several nationwide and complete health data registries allowed for the follow-up of patients for up to 23 years without any losses to follow-up. The quality of the data sources has been extensively validated, and data on medication use and surgical procedures have a particularly high validity.^13,14,17^ The results were adjusted for several potential risk factors, which counteracted confounding. The large cohort size, recruited from 2 countries, provided sufficient statistical power that reduced the risk of random errors and allowed for subgroup analyses.

This study also has some limitations, including the lack of data on some potential confounders (eg, tobacco smoking and body mass index). However, it is unlikely that smoking and obesity were major confounders because both comparison groups consisted of patients with a diagnosis of GERD. Moreover, both these variables were indirectly adjusted for by the Charlson Comorbidity Index.

Antireflux medication combined with secondary antireflux surgery, as a measure of reflux recurrence after fundoplication, is associated with clear benefits because these outcomes largely correlate with symptoms while allowing for a dynamic and complete follow-up. Potential downsides to this definition include misclassification if the indication for resumption was not reflux symptoms. Such misclassification was likely offset in this study by classifying only long-term use of antireflux medication as recurrence because most other indications for antireflux medication require only short-term use (eg, for peptic ulcer disease and *Helicobacter pylori* eradication). Resumption of medication use may also not correlate with physiological studies demonstrating pathologic reflux but should instead be understood as a marker of recurring symptoms. Similarly, secondary antireflux surgery is not always performed for recurring reflux but can be performed for symptoms related to the primary procedure (eg, dysphagia) because of a too-tight fundoplication or recurring hiatal hernia. Although the outcomes studied here largely illustrate recurring symptoms after laparoscopic fundoplication, they should be understood within their limitations. Bearing these limitations in mind, we argue that the findings have strong internal validity and generalizability, at least when applied to countries with comparable health care and demographic characteristics to Finland and Sweden.

## Conclusions

This large population-based cohort study from 2 countries with long and complete follow-up and adjustment for confounders found that laparoscopic fundoplication for nonerosive GERD has rates of reflux recurrence comparable to erosive GERD. This finding contrasts the evidence showing that nonerosive GERD responds less well to antireflux medication. Thus, the absence of erosive GERD during upper endoscopy may not be used as an argument for abstaining from antireflux surgery.

## Notes

Supplement 1. **eTable 1. ***ICD* Codes Used to Define Gastroesophageal Reflux Disease **eTable 2. **Procedure Codes Used to Identify Laparoscopic Fundoplication and Upper Endoscopy **eTable 3. **Procedure Codes Used to Identify Esophageal Procedures Leading to Exclusion **eTable 4. **Procedure Codes Defining Secondary Antireflux Surgery **eTable 5a. ***ICD* Codes Used for Calculating Charlson Comorbidity Score **Table 5b. **Some of the Specified *ICD-9* Codes in Table 5a Correspond to a Different *ICD-9 *Code in Finland; These Are Specified Below **eTable 6. ***ICD* Codes for Oesophageal and Cardia Cancer Used for Censoring
eReferences.

Supplement 2.
Data Sharing Statement
