# Supplementary material for: Reflux Recurrence After Laparoscopic Fundoplication for Nonerosive Gastroesophageal Reflux Disease
Source: JAMA Netw Open. 2025 Jun 30;8(6):e2517754. doi: 10.1001/jamanetworkopen.2025.17754 (PMC12210078; doi:10.1001/jamanetworkopen.2025.17754)
Supplement: Supplement 1. — eTable 1. ICD Codes Used to Define Gastroesophageal Reflux Disease eTable 2. Procedure Codes Used to Identify Laparoscopic Fundoplication and Upper Endoscopy eTable 3. Procedure Codes Used to Identify Esophageal Procedures Leading to Exclusion eTable 4. Procedure Codes Defining Secondary Antireflux Surgery eTable 5a. ICD Codes Used for Calculating Charlson Comorbidity Score Table 5b. Some of the Specified ICD-9 Codes in Table 5a Correspond to a Different ICD-9 Code in Finland; These Are Specified Below eTable 6. ICD Codes for Oesophageal and Cardia Cancer Used for Censoring eReferences. [file jamanetwopen-e2517754-s001.pdf]

Supplementary Online Content

Holmberg D, Bielik J, Santoni G, et al. Reflux recurrence after laparoscopic fundoplication for nonerosive gastroesophageal reflux disease. *JAMA Netw Open.* 2025;8(6):e2517754. doi:10.1001/jamanetworkopen.2025.17754

eTable 1. *ICD* Codes Used to Define Gastroesophageal Reflux Disease
eTable 2. Procedure Codes Used to Identify Laparoscopic Fundoplication and Upper Endoscopy
Table 3. Procedure Codes Used to Identify Esophageal Procedures Leading to Exclusion
eTable 4. Procedure Codes Defining Secondary Antireflux Surgery
eTable 5a. *ICD* Codes Used for Calculating Charlson Comorbidity Score
Table 5b. Some of the Specified *ICD-9* Codes in Table 5a Correspond to a Different *ICD-9* Code in Finland; These Are Specified Below
Table 6. *ICD* Codes for Oesophageal and Cardia Cancer Used for Censoring
eReferences.

This supplementary material has been provided by the authors to give readers additional information about their work.

© 2025 Holmberg D et al. *JAMA Network Open.*

eTable 1. *ICD* Codes Used to Define Gastroesophageal Reflux Disease

	**Sweden**	**Finland**
Gastroesophageal reflux disease	ICD-10: K21.9	ICD-10: K21.9
Hiatal hernia	ICD-8: 551.30<br>ICD-9: 553D<br>ICD-10: K44	ICD-8: 551.30<br>ICD-9: 5513A<br>ICD-10: K44
Heartburn	ICD-8: 784.30<br>ICD-9: 787B<br>ICD-10: R12	ICD-8: 784.30<br>ICD-9: 7871A<br>ICD-10: R12
Esophagitis	ICD-8: 530.94<br>ICD-9: 530B<br>ICD-10: K20, K21.0	ICD-8: 530.94<br>ICD-9: 5301C-D, 5301X<br>ICD-10: K20, K21.0
Barrett's esophagus	ICD-10: K22.7	ICD-9: 5301B<br>ICD-10: K22.7

© 2025 Holmberg D et al. *JAMA Network Open.*

eTable 2. Procedure Codes Used to Identify Laparoscopic Fundoplication and Upper Endoscopy

	**NOMESCO (1997-)**	**Older Swedish classification (before 1997)**	**Older Finnish classification (Toimenpidenimikkeistö, before 1997)**
Laparoscopic fundoplication	JBC01	N/A	N/A
	JBW97	N/A	N/A
Upper endoscopy	UJD*	4480, 4483, 9004	Old classification Finland: 1300, 1310, 1320

© 2025 Holmberg D et al. *JAMA Network Open.*

Table 3. Procedure Codes Used to Identify Esophageal Procedures Leading to Exclusion

	**NOMESCO (1997-)**	**Previous Swedish classification (before 1997)**	**Previous Finnish classification (Toimenpidenimikkeistö, before 1997)**
Oesophageal surgery	JBC*	2800, 2801, 2802	6201-6250<br>6260-6309<br>1301-1304, 1311-1314, 1321-1327
	JBW*	2810, 2811, 2814, 2812, 2813	
	JCA*	2820, 2821, 2822, 2829	
	JCB*	2830	
	JCC*	2840, 2841	
	JCD*	2850, 2851, 2852, 2853, 2854, 2859	
	JCE*	2860, 2861	
	JCF*	2870, 2871, 2872, 2879	
	JCW*	4426, 4430, 4432, 4434, 4435	
Hiatal hernia surgery	JBB00, JBB01, JBB90-93, JBB96, JBB97	4270, 4271, 4272	6251-6259

© 2025 Holmberg D et al. *JAMA Network Open.*

eTable 4. Procedure Codes Defining Secondary Antireflux Surgery

	NOMESCO (1997-)	Previous Swedish classification (before 1997)	Previous Finnish classification (Toimenpidenimikkeistö, before 1997)
Re-do fundoplications (as outcome)	JBC*	N/A	6241-6249
Hiatal hernia surgery	JBB00, JBB01, JBB90-93, JBB96, JBB97	4270, 4271, 4272	6251-6259

© 2025 Holmberg D et al. *JAMA Network Open.*

eTable 5a. *ICD* Codes Used for Calculating Charlson Comorbidity Score

	ICD-8	ICD-9	ICD-10
Country (years of use)[1-5]	Finland (1969-1986) Sweden (1964-1986)	Finland (1987-1995) Sweden (1987-1996)	Finland (1996-) Sweden (1997-)
Myocardial Infarction	410*, 412*	410*, 412*	I21*-I23*, I252*
Congestive Heart Failure	427*, 428*	402*, 425*, 428*, 429D*¥	I11*, I13*, I255*, I42*-I43*, I50*, I517*
Peripheral Vascular Disease	440*- 445*	440*-447*, V43E*¥, 785E*¥	I70*-I73*, I770*-I771*, K551*, K558*- K559*, R02*, Z958*-Z959*
Cerebrovascular disease	430*- 438*	430*-438*, 362D*¥	G45*-G46*, I60*-I69*
Dementia	290*	290*, 294B*¥	A810*, F00*-F03*, F051*, G30*-G31*
Chronic pulmonary disease	490*- 493*, 515*-518*	490*-496*, 500*-505*, 416*, 506E*¥	I26*-I27*, J40*-J47, J60*-J67*, J684*, J701*, J703*
Rheumatic disease	710*-712*, 734*	710*-714*, 725*	M05*-M06*, M09*, M120*, M315*, M32*-M36*
Liver disease	070*, 4560*, 571*, 573*	070*, 571* - 573* 456A*¥-456C*¥	B18*, I85*, I864, I982*, K70*-K71*, K721*, K729*, K76*, R162*, Z944*
Diabetes mellitus	250*	250*	E10*-E14*
Hemiplegia	344*	342*-344*	G114*, G81*-G83*
Renal disease	403* - 404*, 580* - 583*, 792*	403*- 404*, 580-588*, V42A*¥, V45B*¥	I12*-I13*, N01*, N03*, N05*, N07*-N08*, N171*-N172*, N18*, N19*, N25*, Z49*, Z940*, Z992*
Any malignancy	140*- 172*, 174* - 195*, 200*-207*	140*-172*, 174*-195*, 200*-208*	C00*–C26*, C30*–C34*, C37*–C41*, C43*, C45*-C58*, C60–C76*, C80*-C85*, C88*, C90*-C97*
Metastatic tumors	196* - 199*	196*-199*	C77*-C79*
AIDS	N/A	279K*¥	B20*–B24*

© 2025 Holmberg D et al. *JAMA Network Open.*

* All positions that follow are valid without need of further specification.
¥ The corresponding code in Finland is different. Please see Table 5b.

© 2025 Holmberg D et al. *JAMA Network Open.*

Table 5b. Some of the Specified *ICD-9* Codes in Table 5a Correspond to a Different *ICD-9* Code in Finland; These Are Specified Below

	**ICD-9 Finland**
Myocardial Infarction	-
Congestive Heart Failure	429D* → 4293A*
Peripheral Vascular Disease	V43E* → No corresponding code<br>785E* → 7854A*
Cerebrovascular disease	362D* → 3623A*-3623D*
Dementia	294B* → 2941A*
Chronic pulmonary disease	506E → 5064A*
Rheumatic disease	-
Liver disease	456A*- 456C* → 4560A*, 4561A*
Diabetes mellitus	-
Hemiplegia	-
Renal disease	V42A* → No corresponding code<br>V45B* → No corresponding code
Any malignancy	-
Metastatic tumors	-
AIDS	279K* → 0788C*
* All positions that follow are valid without need of further specification.	

© 2025 Holmberg D et al. *JAMA Network Open.*

Table 6. *ICD* Codes for Oesophageal and Cardia Cancer Used for Censoring

	**ICD-10**
Esophageal or cardia cancer	C15, C16.0

© 2025 Holmberg D et al. *JAMA Network Open.*

eReferences.

1. Smedby B, Schiøler G. Health classifications in the Nordic countries. 2006.
2. Schmidt M, Schmidt SA, Sandegaard JL, Ehrenstein V, Pedersen L, Sorensen HT. The Danish National Patient Registry: a review of content, data quality, and research potential. *Clin Epidemiol*. 2015;7:449-90. doi:10.2147/CLEP.S91125
3. Maret-Ouda J, Tao W, Wahlin K, Lagergren J. Nordic registry-based cohort studies: Possibilities and pitfalls when combining Nordic registry data. *Scand J Public Health*. Jul 2017;45(17_suppl):14-19. doi:10.1177/1403494817702336
4. Sund R. Quality of the Finnish Hospital Discharge Register: a systematic review. *Scand J Public Health*. Aug 2012;40(6):505-15. doi:10.1177/1403494812456637
5. och innehåll i patientregistret Utskrivningar K. från slutenvården 1964–2007 och besök i specialiserad öppenvård (exklusive primärvårdsbesök) 1997–2007. *National Board of Health and Welfare*. 2009;

© 2025 Holmberg D et al. *JAMA Network Open.*
