# Supplementary material for: Reflux Recurrence After Laparoscopic Fundoplication for Nonerosive Gastroesophageal Reflux Disease
Source: JAMA Netw Open. 2025 Jun 30;8(6):e2517754. doi: 10.1001/jamanetworkopen.2025.17754 (PMC12210078; doi:10.1001/jamanetworkopen.2025.17754)
Supplement: Supplement 2. — Data Sharing Statement [file jamanetwopen-e2517754-s002.pdf]

Data Sharing Statement

Holmberg. Reflux Recurrence After Laparoscopic Fundoplication for Nonerosive Gastroesophageal Reflux Disease. *JAMA Netw Open*. Published June 30, 2025. doi:10.1001/jamanetworkopen.2025.17754

Data
Data available: No

Additional Information
Explanation for why data not available: Data cannot be shared due to restrictions from the registry holders.
